# Description of *Panagrolaimus namibiensis* n. sp. (Rhabditida: Panagrolaimidae), an Anhydrobiotic Nematode from the Namib Desert of Namibia

**DOI:** 10.2478/jofnem-2024-0039

**Published:** 2024-10-31

**Authors:** Christopher J. Rawson, London Nemmers, Stacey Criswell, Ashleigh B. Smythe, Alison K. Burke, Eugene Marais, Gillian Maggs-Kölling, Amy M. Treonis

**Affiliations:** Department of Biology, University of Richmond, Richmond, VA, 23173; Department of Biology, Virginia Military Institute, Lexington, VA, 24450; Gobabeb Namib Research Institute, Walvis Bay 13013, Namibia; Unit for Environmental Sciences and Management, North-West University, Potchefstroom, South Africa

**Keywords:** Cryptobiosis, morphology, phylogeny, scanning electron microscopy, taxonomy

## Abstract

*Panagrolaimus namibiensis* n. sp. was recovered and cultured from soils collected under *Arthraerua leubnitziae* (pencil-bush) in the Namib Desert of Namibia, one of the driest terrestrial habitats on Earth. It is described here based on morphometrics, scanning electron micrographs, light images, line drawings, and molecular data. The new species is distinguished by having a conspicuous posterior deirid, a hook-shaped stegostomal dorsal tooth, and anterior deirids and excretory pore aligned at mid-bulb. It was morphologically compared to eleven well-described species in the genus with which it shared similar labial structure (six distinct rounded lips, and low lip segments separated in pairs), conoid tail, and/or a lateral field with three incisures, including *P. labiatus*, *P. kolymaensis*, *P. davidi*, *P. rigidus*, and *P. superbus*. Bayesian phylogenetic analyses using SSU and LSU rDNA each placed *P. namibiensis* n. sp. within clades of *Panagrolaimus* species, although the two trees resolved its relationship to previously described species differently. Furthermore, our analyses showed the genus is not monophyletic. In a laboratory experiment, *P. namibiensis* n. sp. survived exposure to 0% relative humidity for 24 h, demonstrating the anhydrobiotic ability of this species that contributes to its survival in the Namib Desert.

*Panagrolaimus* Fuchs, 1930 is a globally distributed genus of bacterial-feeding nematodes from the family Panagrolaimidae Thorne, 1937 that contains species associated with detritus (Andrássy, 2005) or arthropods (Hodda, 2022). They are distinguished, among other features, by having six flat lips, a pipe-shaped stoma with a minimally sclerotized cheilostom, and a pharyngeal corpus somewhat swollen at the base and distinctly separate from the isthmus. At least forty species of *Panagrolaimus* have been described (Andrássy, 2005, Abolafia and Peña-Santiago, 2006), but the limited morphological variation amongst *Panagrolaimus* spp. makes them difficult to delineate (Abebe and Blaxter, 2003). Species have been described from diverse terrestrial habitats including gulls’ nests on the island of Surtsey, Iceland (Boström 1988), upland grassland soils in the U.K. (Abebe and Blaxter, 2003), leatherleaf slugs in India (*Laevicaulis* sp., Yadav et al., 2023), and volcanic soils on Ross Island, Antarctica (Timm, 1971). These nematodes also have been found in meiobenthic communities (Derycke et al., 2007; De Oliveira Pinto et al., 2021).

*Panagrolaimus* nematodes are model organisms for the study of animal survival strategies in extreme environments (Aroian et al., 1993; Wharton 1998; Shannon et al., 2005; Schiffer et al., 2019; Shatilovich et al., 2023). They can enter an ametabolic state of cryptobiosis (Keilin 1959), allowing them to persist when water is not biologically available due to temperature (cryobiosis) or desiccation (anhydrobiosis). *Panagrolaimus superbus* Fuchs, 1930 has been the subject of a plethora of *in vitro* studies describing its outstanding ability to survive extreme desiccation in anhydrobiosis (Shannon et al., 2005; de Souza et al., 2017). Recently, Shatilovich et al. (2023) revived cryobiotic nematodes, including *Panagrolaimus kolymaensis* Shatilovich et al., 2023, from 46,000-year-old permafrost samples. Other *Panagrolaimus* species have similarly been revived from cryptobiosis induced by exposure to desiccation and freezing (McGill et al., 2015). Cryptobiosis appears to be a widespread strategy in *Panagrolaimus* and is likely to contribute to the broad distribution of these organisms (Treonis and Wall, 2005; Shatilovich et al., 2023).

The Namib Desert of Namibia, in Southern Africa, is a narrow desert that extends along the entire Atlantic coast of the country. Parts of the Namib Desert consist of vast dune systems, while others consist of relatively barren gravel plains. Both systems are extremely arid (< 100 mm annual rainfall) and receive moisture from coastal fogs as well as sporadic rains, resulting in unique, fog-based ecologies for many organisms (Mitchell et al., 2020). Several novel nematode species were described from the Namib Desert decades ago (Rashid et al., 1990a, 1990b; Rashid and Heyns, 1990a, 1990b), but no Panagrolaimidae were included. More recent studies have employed morphological and molecular approaches to study the composition of Namib Desert soil nematode communities (Marais et al., 2020; Treonis et al., 2022, 2024). Each of these studies has noted the presence of *Panagrolaimus* species. For example, using a metabarcoding approach, Marais et al. (2020) found that most soil samples collected in the Namib Desert sand dunes from under *Acanthosicyos horridus* Welw. ex Hook. f. (!nara melon) contained 18S sequences assigned to *Panagrolaimus*. Furthermore, a *Panagrolaimus* sp. was often numerically dominant in soil nematode communities under shrubs and *Welwitschia mirabilis* Hook. f. plants in the Namib Desert gravel plains (Treonis et al., 2022, 2024). The apparent wide distribution of *Panagrolaimus* spp. in the Namib Desert supports the need to identify the species and to study its survival strategies in this extreme arid environment.

Here, a new species of *Panagrolaimus* from the Namib Desert is described through morphological and molecular phylogenetic analyses. This nematode was cultured in the laboratory, and we also performed *in vitro* assays to assess its ability to enter anhydrobiosis and survive extreme desiccation.

## Materials and methods

### Study site & sampling

**Figure 1:**
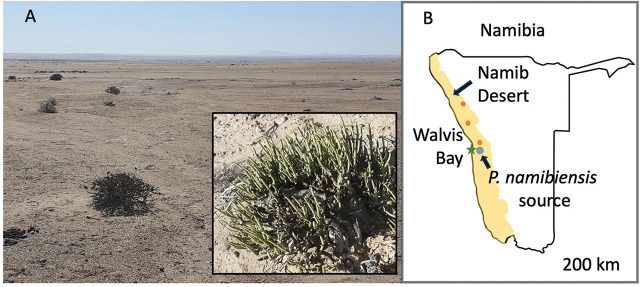
A) Soil collection site for isolation of *Panagrolaimus namibiensis* n. sp. Soils were collected from under *Arthraerua leubnitziae* shrubs (inset). B) Map of Namibia showing location of soil collection site (blue dot). Red dots indicate other soils containing a *Panagrolaimus* nematode morphologically indistinct from *P. namibiensis* n. sp.

Soil samples were collected in the Namib Desert from beneath a common shrub, pencil-bush, *Arthraerua leubnitziae* (Kuntze) Schinz (Fig. 1). Soils were collected using plastic scoops from multiple locations under the shrub branches from a depth of 0 to 10 cm (litter was gently scraped to the side first). Soils were sieved in the field (2 mm mesh) to remove rocks and transferred to plastic bags. Samples were transported to the University of Richmond (Virginia, USA) and stored at 4°C. Nematodes were extracted over 72 h from soil using a Baermann funnel technique (Baermann, 1917) and subsequently transferred to uninoculated baby food agar (BFA) plates (Stock et al., 2001). Nematodes, and the bacteria they brought with them, flourished on the plates. From this initial culture, a subsequent culture was established from a single female nematode that was transferred to a new plate using a sterile platinum wire pick.

### Sanitization

To sanitize the culture, we employed a 36-h surface sterilization technique (modified from Manzanilla-López and Ehlers, 2021). Nematodes were washed from culture plates with deionized water and pipetted into warm 1% water agar at the bottom of a petri dish. A second warm layer of 1% water agar containing antibiotic (0.3%, dihydrostreptomycin) and antifungal (0.03%, amphotericin B) solutions was added for the nematodes to migrate through over the ensuing 36 hours. The nematodes on the top of the plate were then aseptically washed onto a new BFA plate that was pre-inoculated with an 18 to 24 h broth of *Escherichia coli* OP50 (Brenner, 1974) in Tryptic Soy Broth. Plates were maintained at 15°C.

### Scanning electron microscopy

Nematodes were washed into a microcentrifuge tube with deionized water. Centrifugation was performed after each processing step to ensure maximum retention of the nematode sample. The nematodes were fixed in a cocktail of 4% paraformaldehyde and 2% glutaraldehyde in sodium cacodylate buffer, followed by a secondary fixation in 1% osmium tetroxide, and subsequently run through serial ethanol dehydration. The final suspension of nematodes in 100% ethanol was then placed in a styrofoam basket where the nematodes were critical-point dried, mounted, and sputter-coated. Images were taken using a JEOL JSM-IT700HR scanning electron microscope (JEOL USA, Inc., Peabody, MA). Cultured nematodes were induced into anhydrobiosis in relative humidity chambers on Supor^®^ membrane filters (Pall Corporation, Port Washington, NY) and then directly sputter-coated and imaged.

### Light microscopy

Nematodes were fixed with 5% formalin-glycerin (Seinhorst, 1962), mounted on temporary glass slides, and examined on a Zeiss Axiovert microscope under oil with a x63 ocular lens and a x1.6 Optovar cube to achieve x1000 magnification. Digital measurements of nematode characters were performed using ZEN 2.3 software (Carl Zeiss, Inc., White Plains, NY). Light images were collected using a Nikon Eclipse Ci DIC-equipped compound microscope (Nikon Instruments, Tokyo, Japan) and an Olympus Fluoview FV1200 equipped with a Q Imaging scientific CMOS camera (Olympus Microscope, Tokyo, Japan).

### Line drawings

Line drawings were performed by digitally tracing the superimposition of multi-focal plane light images.

### Molecular phylogenetic analyses

DNA was extracted from a whole, single, individual male and female of *P. namibiensis* n. sp. using the DNeasy Blood and Tissue Kit (Qiagen Inc., Valencia, CA) according to the manufacturer’s instructions. An approximately 700 base-pair fragment of the 5′ end of the 28S nuclear ribosomal large subunit (LSU), including the D2-D3 variable domains, was amplified and sequenced using forward primer Pd2a (5′ACAAGTACCGTGAGGGAAAGT3′) (Kelly Thomas, UNH, pers. comm.) and reverse primer D3B (5′TGCGAAGGAACCAGCTACTA3′) (Nunn, 1992). An approximately 600 base pair fragment of the 5′ end of the 18S nuclear ribosomal small subunit (SSU) was amplified and sequenced using forward primer pNem18SF (5′CGCGAATRGCTCATTACAACAGC3′) and reverse primer pNem18SR (5′GGGCGGTATCTGATCGCC3′) (Floyd et al., 2005). Polymerase chain reactions (PCR, 25 μl total reaction volume) were performed using 2 to 3 μl of DNA template, 12.5 μl of DreamTaq Green PCR master mix (ThermoFisher Scientific, Waltham, MA) and 1.5 μl of each primer at 10 μM concentration. The PCR thermocycling parameters included denaturation at 94°C for 5 min, followed by 35 cycles of 94°C for 30 s, 50 to 51°C (LSU) or 54°C (SSU) for 30 s, and 72°C for 1 min. A final extension period of 5 min at 72°C concluded the amplification. Prior to direct sequencing, PCR products were enzymatically treated with ExoSAP-It PCR Product Cleanup Reagent (ThermoFisher Scientific, Waltham, MA) to remove excess primers and dNTPs. Sequencing reactions were conducted by GENEWIZ/Azenta Inc. (South Plainfield, NJ) with the original PCR primers. Geneious Prime 2023.1.2 (https://www.geneious.com) was used for examination of electropherograms and contig assembly. Sequences obtained for the male and female *Panagrolaimus namibiensis* n. sp. have been deposited in GenBank as accession numbers PP692155, PP692156, PP69763, and PP697634.

Taxon inclusion for the LSU and SSU analyses aimed to match those included in Yadav et al. (2022) and Shatilovich et al. (2023), though some sequences were excluded due to being considerably shorter than the fragment sequenced for *P. namibiensis* n. sp. For both the LSU and SSU analyses, sequences of *Acrobeles complexus* Thorne 1925 (Cephalobidae Filipjev, 1934) and *Caenorhabditis elegans* (Maupas 1900) Dougherty 1953 N2 strain (Rhabditidae Örley, 1880) were included as outgroups. The SSU analysis included 63 total sequences, with two sequences representing a male and female of *P. namibiensis* n. sp. (Supplementary Table 1). Sequences were downloaded from GenBank that met the following criteria: unique (not identical), at least 500 base pairs in length, and including the 5′ end of the SSU locus. Forty-two published SSU sequences of *Panagrolaimus* spp., ten representatives of all available genera of Panagrolaimidae [as listed in the Nemys database (Nemys eds., 2024)], and one unidentified member of Panagrolaimidae (isolate DS129) were included in the alignment and phylogenetic analysis. Three members of Alloionematidae Chitwood & McIntosh, 1934, two members of Brevibuccidae Paramonov, 1956, and one member of Strongyloididae Chitwood & McIntosh, 1934 were also included. The LSU analysis included 36 total sequences in the alignment and phylogenetic analysis (Supplementary Table 1), again with two sequences representing a male and female of *P. namibiensis* n. sp. Thirty-four published LSU sequences were downloaded from GenBank that were unique, had at least 600 base pairs in length, and that included the D2-D3 variable domain. All the available sequences of *Panagrolaimus* (21) that met these criteria were included, as well as the same unidentified member of Panagrolaimidae (isolate DS129). Sequences from eleven members of other genera that met these criteria were also included: representatives of all available genera of Panagrolaimidae (Nemys eds., 2024), three members of Alloionematidae, two members of Brevibuccidae, and one member of Strongyloididae.

**Table 1:** Measurements of *Panagrolaimus namibiensis* n. sp. from the gravel plains of the Namib Desert of Namibia. Measurement units are μm ± the standard deviation, with the range in parentheses.

**Character**	**Females**		**Males**

	**Holotype**	**Paratypes**	**Paratypes**
n	-	12	13
L	900.8	897.8 ± 44.9 (831.5–976.7)	764.6 ± 49.3 (717.7–853.0)
a	22.0	21.1 ± 3.0 (16.5–25.2)	22.0 ± 1.9 (18.0–25.3)
b	5.9	6.2 ± 0.4 (5.7–7.0)	5.7 ± 0.3 (5.2–6.3)
c	17.5	21.5 ± 1.3 (19.3–23.6)	19.5 ± 1.6 (17.5–22.4)
c’	1.9	1.8 ± 0.2 (1.4–2.1)	1.6 ± 0.2 (1.3–1.9)
V (%)	60.9	59.3 ± 1.5 (57.2 – 61.6)	-
Lip region width	8.9	8.5 ± 0.5 (7.7–9.3)	7.5 ± 0.5 (7.0–8.6)
Stoma length	11.4	12.1 ± 0.8 (10.9–13.3)	11.5 ± 0.9 (10.4–12.8)
Stoma width	2.2	2.4 ± 0.2 (2.1–2.8)	2.2 ± 0.2 (1.9–2.7)
Corpus length	89.7	88.3 ± 3.4 (81.7–92.6)	79.9 ± 5.6 (69.8–90.9)
Corpus width	19.7	19.7 ± 1.8 (17.5–23.6)	15.8 ± 2.6 (12.4–21.1)
Isthmus	27.7	28.9 ± 2.0 (25.3–32.6)	29.0 ± 1.9 (25.5–32.9)
Basal bulb	27.2	27.1 ± 2.1 (24.8–31.3)	24.7 ± 2.3 (22.5–29.4)
Pharynx length	144.6	144.9 ± 4.9 (135.2–152.2)	133.6 ± 6.8 (123.9–149.0)
Neck	152.8	154.8 ± 6.1 (145.9–166.1)	140.6 ± 7.2 (131.0–155.9)
Neck-base body diameter	36.4	38.7 ± 4.6 (32.9–47.8)	33.2 ± 4.5 (25.0–40.4)
Nerve ring - ant. end	115.0	115.3 ± 7.1 (102.2–127.4)	105.7 ± 9.1 (91.7–123.2)
Excretory pore - ant. end	141.0	141.1 ± 11.3 (115.1–157.6)	136.0 ± 12.2 (115.4–157.0)
Deirid - ant. end	141.0	141.7 ± 11.3 (115.4–157.1)	136.5 ± 12.6 (115.4–157.6)
Mid-body diameter	40.9	43.4 ± 5.8 (34.6–55.4)	35.1 ± 4.2 (29.5–41.9)
Cuticle annuli thickness	1.2	1.2 ± 0.1 (1.1–1.4)	1.2 ± 0.1 (1.0–1.4)
Lateral field	3.7	3.7 ± 0.4 (3.2–4.3)	3.5 ± 0.4 (3.1–4.2)
Vulva - ant. end	548.5	532.3 ± 26.5 (499.0–572.8)	-
Vulval body diameter	41.6	44.1 ± 5.1 (35.9–50.5)	-
Vulva-anus distance	298.2	302.6 ± 14.8 (275.2–321.1)	-
Vagina length	13.7	14.2 ± 1.5 (11.3–16.5)	-
Ovary length	342.3	389.7 ± 30.0 (326.8–430.3)	-
Post-vulval sac	25.5	21.9 ± 3.4 (16.5–27.2)	-
Rectum length	27.9	25.8 ± 2.8 (20.1–30.3)	-
Testes length	-	-	207.8 ± 22.3 (184.1–254.9)
Spicule length	-	-	29.3 ± 2.0 (24.5–31.6)
Gubernaculum length	-	-	12.6 ± 1.5 (10.9–15.4)
Tail	51.4	42.3 ± 2.3 (39.2–45.9)	39.4 ± 3.6 (32.5–45.9)
Phasmid-anus distance	23.1	18.5 ± 3.5 (13.3–24.2)	18.9 ± 3.7 (14.3–28.7)
Anal body diameter	26.8	23.4 ± 2.9 (20.4–31.0)	24.7 ± 2.9 (20.8–29.3)

To obtain the nuclear ribosomal operon of *P. kolymaensis*, the genome assembly (GCA_028622995.1) was downloaded from NCBI and queried using publicly available *Panagrolaimus* sequences that collectively span most of the nuclear ribosomal operon (NCBI Nucleotide database accession numbers EU253569.1, LT837699.1, DQ285636.1) and the complete nuclear ribosomal operons of three other nematodes (*Meloidogyne chitwoodi* Golden, O’Bannon, Santo & Finley 1980 - ON496983.1, *Longidorus macrosoma* Hooper 1961 - AY580055.1, and *Xiphinema americanum* (*sensu stricto*) Cobb, 1913 - AY580056.1) with blastn 2.9.0+ with an e-value cutoff of 1e-100. The top ten hits according to bitscore (ranging from 4667 to 4673) were aligned to the query sequences using mafft 7.453 using the --ep 0 --genafapair options. These sequences exhibited low intragenomic variation, and thus a representative sequence was selected haphazardly.

For both the LSU and SSU analyses, multiple alignments were conducted using Clustal Omega 1.2.3 (Sievers et al., 2020) as implemented in Geneious Prime 2023.1.2 (https://www.geneious.com) using default parameters. MrBayes 3.2.7a x86_64 (Ronquist et al., 2012) was used for Bayesian inference of phylogenetic trees using the CIPRES Science Gateway portal (http://www.phylo.org). The default setting of the “4by4” nucleotide model was used as recommended by the manual for non-coding DNA sequences (Ronquist et al., 2020). Mr. Bayes allows the Markov chain Monte Carlo to sample across the entire general time-reversible (GTR) substitution model space (“Nst=mixed” command), rendering *a priori* model testing unnecessar y (Ronquist et al., 2020). This also allows the program to use a weighted average of the posterior probability of the best substitution models rather than using a single model (Ronquist et al., 2020). Default, uninformative prior probabilities were used for stationary state frequencies and substitution rates. Each analysis ran for ten million generations as that provided better chain convergence than one million or fewer generations, and the chains were sampled every 2,000 generations with burn-in set to 25%. Support for internal clades was shown by posterior probability (pp) values.

### Induction of anhydrobiosis

Nematodes used for anhydrobiosis experiments were taken from 10-day laboratory cultures. Nematodes were washed from culture plates using 1 ml sterile M9 buffer (Brenner, 1974), transferred to a glass tube, and vortexed gently to evenly distribute the nematodes. An aliquot of this solution containing approximately 100 to 300 nematodes was then transferred to the surface of a 0.45 µm Supor^®^ membrane filter (de Souza et al., 2017) on top of a 50-mm bottle-top Nalgene™ filter apparatus (Thermo Fisher Scientific, Waltham, MA). Gentle suction was applied to remove excess buffer solution. Immediately after vacuum filtration, membrane filters were each placed in a 50-mm petri dish base and transferred to a 100% relative humidity (RH) chamber for 24 hours. RH chambers consisted of 8-inch diameter airtight glass desiccators within which target RH levels were established (see below).

After an initial 24 h at 100% RH, nematodes on filters were subjected to one of the following three treatments. First, filters from a baseline treatment were immediately resuspended in 3 ml M9 buffer and examined to establish nematode viability at the start of the experiment. Second, after 24 h at 100% RH, a preconditioned treatment was moved to 97% RH. The nematodes remained at 97% RH for 72 h, were transferred to 85% RH for 24 h, and then to 75% RH for 24 h. After 24 h at 75% RH, the preconditioned nematodes were transferred to a desiccator at 0% RH for 24 h. Third, the direct exposure treatment was immediately transferred to 0% RH after 24 h at 100%. After 24 h at 0% RH, the preconditioned and direct exposure treatment nematodes were rehydrated by adding 3 ml M9 buffer to each petri dish, which were then transferred to 100% RH. Following 24 h of rehydration, the percentage of nematodes on each filter that were alive (regained motility) and dead (non-motile) was determined and used to calculate a percent survival. A 395 nm UV light was periodically flashed at the filter to stimulate motility of the worms (Rajasekharan et al., 2018). Each of the three treatments (baseline, preconditioned, and direct exposure) was replicated eight times, with the filters containing an average of 171 ± 32 nematodes.

One hundred percent RH was maintained by placing two 60-ml beakers of deionized water in the chambers. Other RH levels were achieved using 60 ml of saturated salt solutions (potassium sulfate for 97% RH, potassium chloride for 85% RH, and sodium chloride for 75% RH) or 5 g phosphorus pentoxide for 0% RH (Winston and Bates, 1960; Solomon et al., 1999). The efficacy of each salt solution at achieving and maintaining the desired RH was monitored using a hygrometer (Traceable^®^, Cole-Parmer, Vernon Hills, IL). All RH chambers were established with fresh solutions for 24 h before any nematodes were added, and they were incubated at 25°C throughout the experiment.

### Statistical analyses

Statistical analyses were performed with R version 4.3.1 (https://www.r-project.org) (R Development Core Team, 2023). Analysis of variance (ANOVA) was used to study differences in nematode survival among the treatment groups. Where effects were significant, means were compared using Tukey’s Honestly Significant Difference (HSD) multiple comparison procedure. Prior to ANOVA, the Shapiro-Wilk test was used to assess the percent survival for normality, and percent survival was arcsine (sqrt(x/100)) transformed.

## Results

### *Panagrolaimus namibiensis* n. sp.

Figs. 2–4.

Measurements, see Table 1.

**Figure 2:**
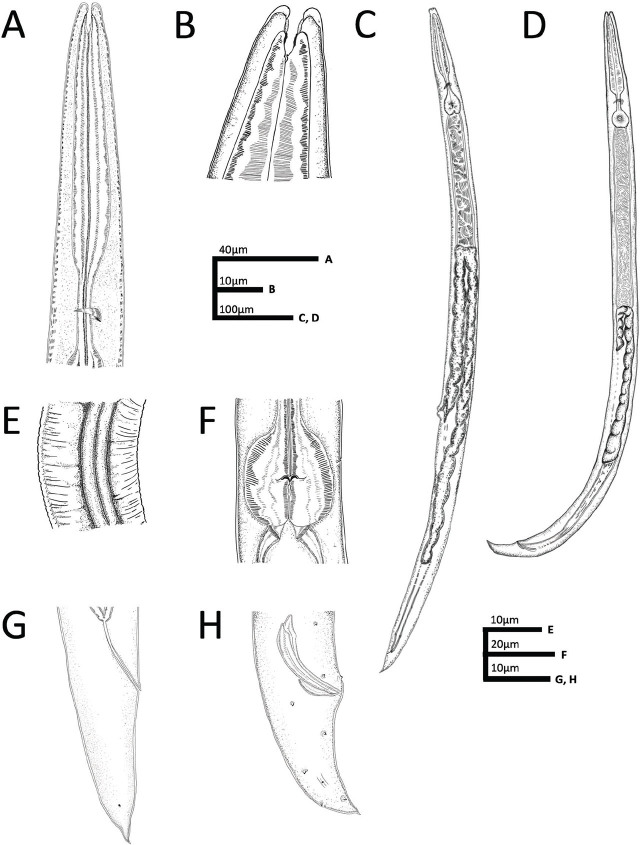
*Panagrolaimus namibiensis* n. sp. line drawings. A: Anterior body region. B: Anterior body end, showing stoma and rhabdia. C: Full body, female. D: Full body, male. E: Lateral field. F: Basal bulb, excretory pore. G: Posterior body end, female. H: Posterior body end, male.

**Figure 3:**
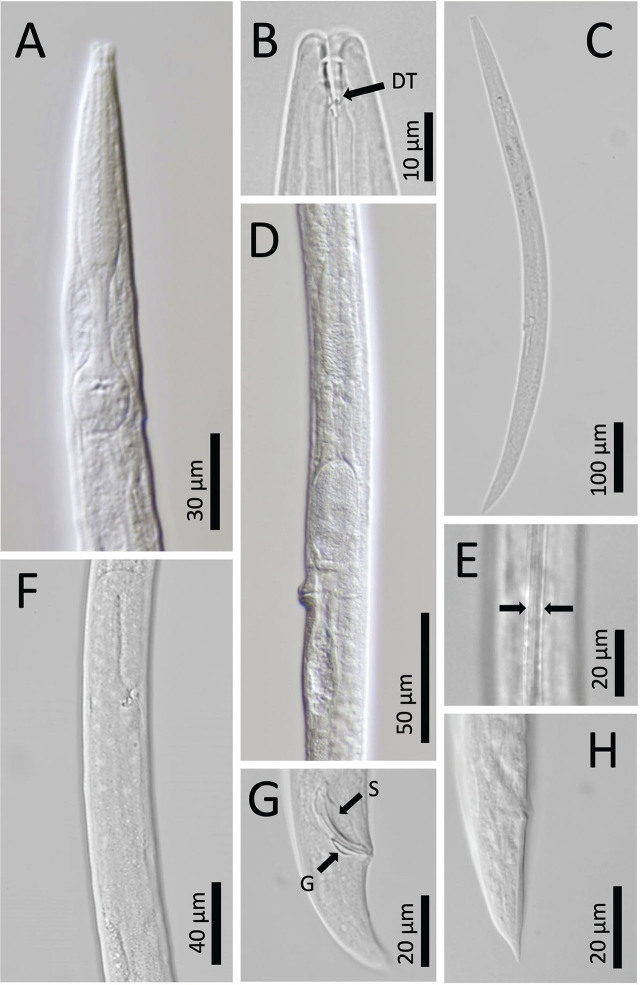
*Panagrolaimus namibiensis* n. sp. light micrographs. A: Anterior body region and pharynx. B: Lip region, stoma, stegostomal dorsal tooth (DT). C: Full body, female. D: Part of reproductive tract, female. E: Lateral field. F: Part of testis, male. G: Posterior body end, spicule (S), gubernaculum (G), male. H: Posterior body end, rectum, female.

**Figure 4:**
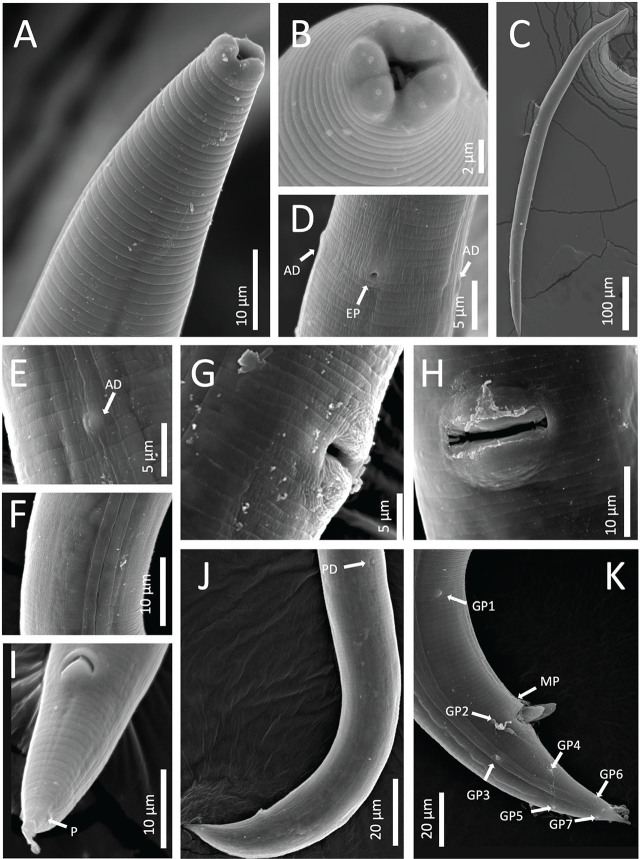
*Panagrolaimus namibiensis* n. sp. scanning electron microscopic images. A: Anterior body region. B: Lip region in frontal view. C: Full body, female. D: Excretory pore (EP), anterior deirids (AD). E: Anterior deirid (AD). F: Lateral field. G, H: Vulva. I: Anus, phasmid (P), female. J: Posterior end, posterior deirid (PD), female. K: Posterior end, spicule, pre- and post-cloacal genital papillae (GP), midventral papillae (MP), male.

## Description

### Adult specimens (sex-independent)

Body straight, cylindrical, ventrally curved (greater in males) post-fixation. Cuticle finely annulated, thickest at mid-body. Prominent lateral fields containing two distinct raised bands (three incisures) with gradual post-cloacal and prephasmid taper. Lip region thinly cuticularized with six distinct rounded lip segments separated in pairs. Labial papillae pronounced. Amphid apertures inconspicuous. Stoma wide, pipe-like, panagrolaimoid, containing distinctive cheilostom, gymnostom, and stegostom. Both cheilostom and gymnostom contain cuticularized rhabdia, with gymnostom also being heavily sclerotized. Minute, hooked dorsal tooth protruding from stegostom. Pharyngeal sleeve conspicuous but variable in stoma overlap, covering 21 to 60% of total stoma length. Pharyngeal corpus cylindrical with long procorpus and marginally prominent, shorter metacorpus. Isthmus long and thin, clearly distinct from metacorpus. Nerve ring near midpoint of isthmus. Anterior deirids in line with excretory pore, lying toward terminus of pharynx at mid-bulb level. Basal bulb swollen and generally spheroid, with noticeable pharyngo-intestinal valve. Cardia conoid, surrounded by thick intestinal tissue. Body widest at mid. Conspicuous posterior deirid dorsal to lateral field and posterior to mid-body. Base of tail wide at anus, narrowing sharply past phasmids.

#### Female

Reproductive system monodelphic-prodelphic. Vulva posterior to mid-body with large, protruding lips. Spermatheca large, ovoid, offset. Oviduct tubular. Uterus wide close to vagina, narrowing toward anterior body end. Post-uterine sac distinct with variable length. Vagina inset at 31 to 33% of vulval body diameter. Rectum 97 to 98% of anal body diameter. Phasmids distinct at 33 to 65% of tail length from posterior end. Tail conoid with pointed terminus.

#### Male

Reproductive system monorchic, testis reflexed anteriorly. Gubernaculum well-developed, 42 to 49% of spicules length. Spicules slightly ventrally curved with well-developed manubrium, inconspicuous calamus and blunt terminus. Genital papillae include two pre-cloacal pairs (GP) (both subventral), one midventral (MP), and five post-cloacal comprising one lateral, two dorsal, and two ventral pairs (GP). Tail conical, slightly ventrally bent with acute tip. Phasmids visible in most specimens.

### Etymology

*P. namibiensis* n. sp. is named for the country in which it was found.

### Type locality and habitat

*Panagrolaimus namibiensis* n. sp. was collected from the Namib Desert gravel plains in Namib Naukluft National Park, Namibia, Africa from soils under the shrub *Arthraerua leubnitziae* (pencil-bush), GPS: 22°56′35.2″S 14°54′51.9″E.

### Type materials

Eight slides were deposited in the Nematode Collection at the United States Department of Agriculture, Beltsville, Maryland, USA, including a female holotype (slide T-799t) and paratypes (three females, slides T-8028p, T8039p, T-8030p, and four males, slides T-800t, T8031p, T8032p, T8033p). The LSID code of this publication is: urn:lsid:zoobank.org:pub:9FF9A841-DB31-4D51-9542-8BE9C991B833.

### Diagnosis and relationships

Main characteristics of *P. namibiensis* n. sp. include the presence of a hook-shaped stegostomal dorsal tooth and a distinct posterior deirid in both males and females. Additionally, the excretory pore is aligned with both anterior deirids and the middle of the basal bulb. Based on sharing morphological features [e.g., structure of the lip region (six distinct rounded, low lip segments separated in pairs), number of lateral incisures (three), and/or general tail shape (conoid)], the new species resembles *Panagrolaimus artyukhovskii* Blinova and Mishina, 1975, *Panagrolaimus conophthori* Massey, 1974, *Panagrolaimus davidi* Timm, 1971, *Panagrolaimus detritophagus* Fuchs, 1930, *Panagrolaimus goodeyi* Rühm, 1956, *P. labiatus* (Kreis, 1929) Andrássy, 1960, *Panagrolaimus leperisini* Massey 1974, *Panagrolaimus orientalis* Korenchenko, 1986, *Panagrolaimus rigidus* Schneider, 1866, *Panagrolaimus subelongatus* Cobb, 1914, and *P. superbus*. Based on phylogenetic analyses (see below), *P. namibiensis* n. sp. is most closely related to *P. labiatus*, *P. kolymaensis, P. rigidus,* and *P. davidi*. The new species can be distinguished from these morphologically similar and phylogenetically related species as follows:

From *P. artyukhovskii* by females having a longer body (717.7–976.2 vs. 710–770 μm), males having two pre-cloacal papillae within the range of the spicules (vs. one) and lack of dentiform thickenings in the metastom.

From *P. conophthori* by having labial papillae (absent in *P. conophthori*), three lateral incisures (vs. four), shorter average female length (897.8 vs. 960 μm), and absence of metarhabdion denticles.

From *P. davidi* by having a shorter average male reproductive tract (207.8 μm vs. 450 μm), a hooked-shaped tooth vs. a triangular tooth, eight total pairs of male genital papillae (vs. six), and two pre-cloacal pairs of genital papillae (vs. one).

From *P. detritophagus* by having lips fused in pairs (unfused lips in *P. detritophagus*), excretory pore opposite the basal bulb (vs. near the middle of the isthmus), and eight pairs of genital papillae (vs. seven).

From *P. goodeyi* by having a shorter cheilogymnostom relative to width (less than two times the width vs. greater than three times the width).

From *P. kolymaensis* by gonochorism as indicated by the presence of males in both laboratory cultures and field samples (*P. kolymaensis* is parthenogenic).

From *P. labiatus* by having a stegostomal dorsal tooth (absent in *P. labiatus*), an excretory pore at basal-bulb level (vs. the middle of the isthmus), and a broad tail lacking acute posterior elongation.

From *P. leperisini* by having rounded labial papillae that lack labial or cephalic setae (present in *P. leperisini*) and an excretory pore at basal-bulb level (vs. opposite the nerve ring).

From *P. orientalis* by having inconspicuous amphidial apertures (conspicuous in *P. orientalis*), a cuticularized cheilostom containing distinct rhabdia (absent in *P. orientalis*), a longer female body (717.7–976.2 vs. 500–700 μm), a non-elongated conical tail, and a non-rounded manubrium.

From *P. rigidus* by having a dorsal tooth, nonsetose labial papillae, a generally broader frame [e.g. a wider average lip width (8.5 vs. 5.7 μm), a wider average mid-body diameter (43.4 vs. 32.3 μm), and a wider anal-body diameter (23.4 vs. 17.5 μm)], and an excretory pore aligned with both anterior deirids and the middle of the basal bulb (vs. the anterior end of the bulb).

From *P. subelongatus* by having six distinctly segmented lips (vs. three), a significantly longer body in females (717.7–976.2 vs. 600–680 µm), and an excretory pore aligned with anterior deirids at the middle of the basal-bulb (vs. deirids and excretory pore near the anterior end of the bulb).

From *P. superbus* by shorter average female body length (897.8 vs. 1200 μm), and two pre-cloacal and one midventral genital papillae (vs. three pre-cloacal).

### Molecular analyses and phylogeny

**Figure 5:**
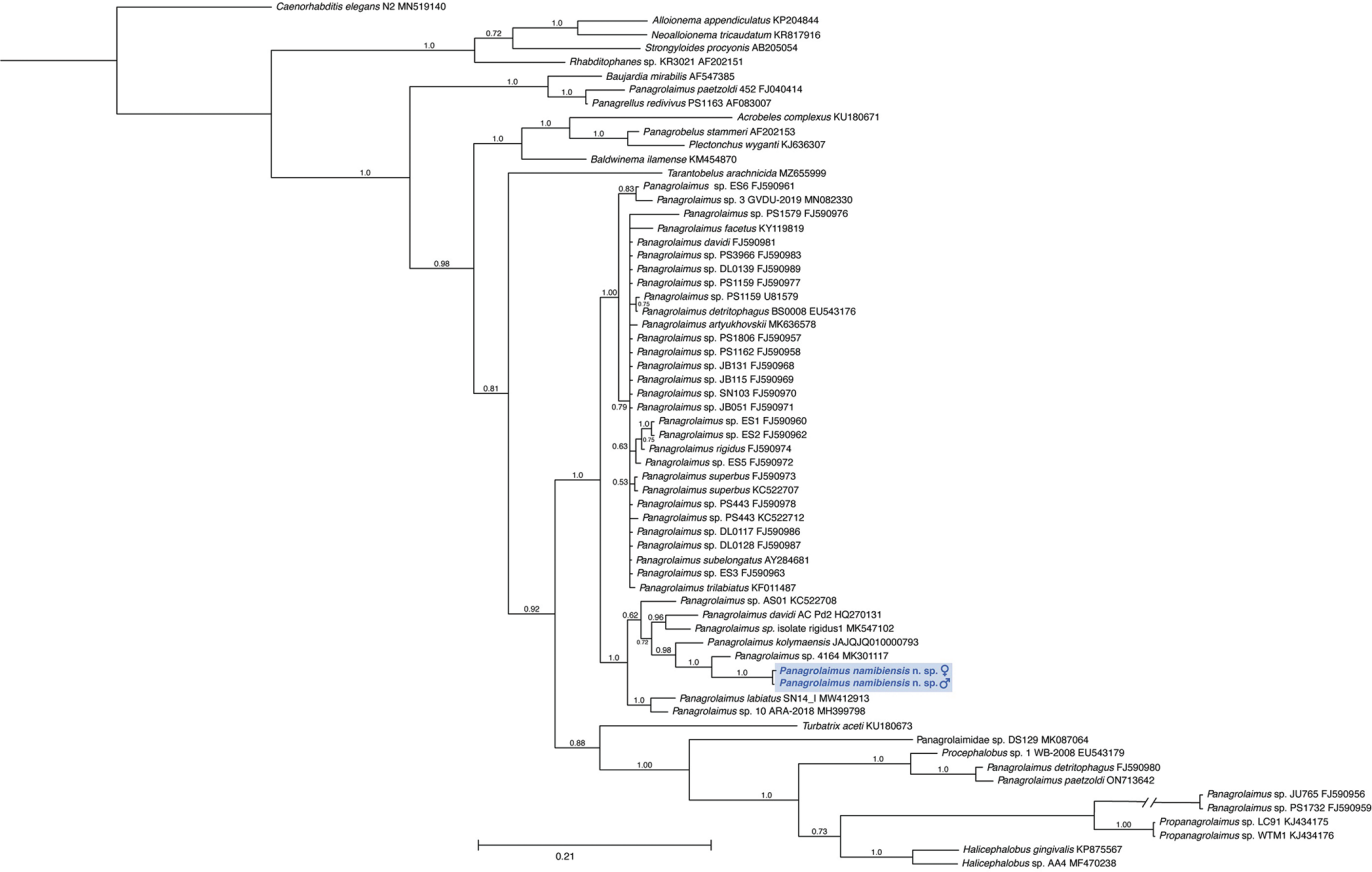
Phylogenetic tree inferred by Bayesian analysis of SSU sequences showing the relationship of *Panagrolaimus namibiensis* n. sp. (highlighted in blue) to other members of Panagrolaimidae and other taxa. Support for internal clades is shown by posterior probability (pp) values placed next to nodes. Scale bar represents 0.21 estimated substitutions per site.

**Figure 6:**
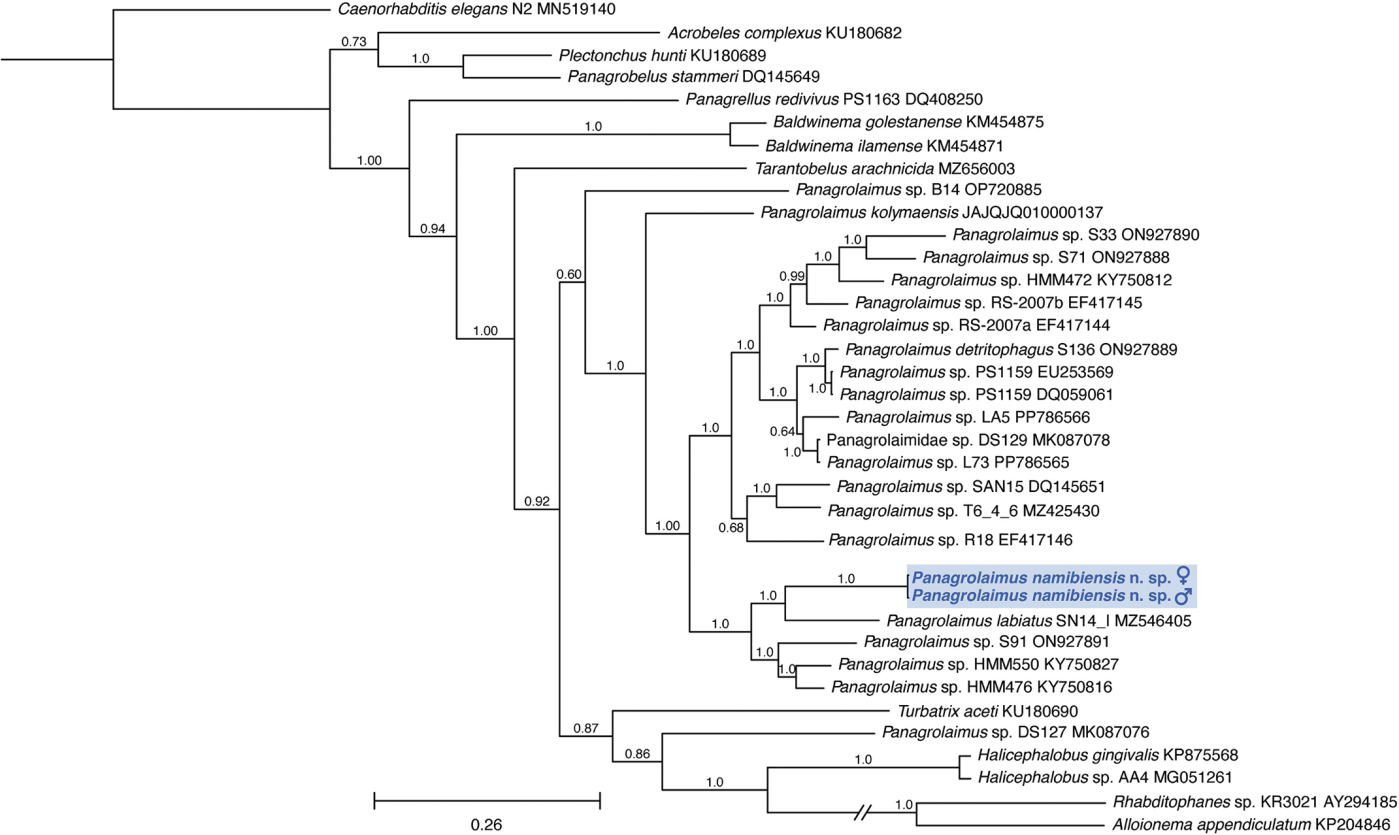
Phylogenetic tree inferred by Bayesian analysis of LSU sequences showing the relationship of *Panagrolaimus namibiensis* n. sp. (highlighted in blue) to other members of Panagrolaimidae and other taxa. Support for internal clades is shown by posterior probability (pp) values placed next to nodes supported by greater than 0.5 pp. Scale bar represents 0.26 estimated substitutions per site.

Both the SSU and LSU phylogenetic analyses confirmed the placement of *P. namibiensis* n. sp. within the Panagrolaimidae in strongly supported clades consisting of most of the *Panagrolaimus* species that were included in the analyses (Figs. 5 and 6). In the SSU analysis, *P. namibiensis* n. sp. was placed with full support (1.0 pp) as sister taxon to an undescribed species, *Panagrolaimus* sp. 4164 from an unknown location (Holterman et al., 2019) (Fig. 5). The clade formed by *Panagrolaimus* sp. 4164 and *P. namibiensis* n. sp. was well supported (0.98 pp) as sister to *P. kolymaensi*s from Siberia (Shatilovich et al., 2023). More broadly, *P. namibiensis* was shown to be part of a maximally supported (1.0 pp) clade that included one of the included sequences of *P. davidi* (isolate AC Pd2), *Panagrolaimus* sp. isolate rigidus1, and *P. labiatus*. Twenty-seven members of *Panagrolaimus* were part of a moderately supported (0.79) clade that was nearly completely unresolved. While the majority of included members of *Panagrolaimus* (36 out of 42) formed a strongly supported clade (1.0 pp), the genus was shown to be non-monophyletic, as several members formed additional clades with taxa like *Turbatrix aceti* (Müller, 1783) Peters, 1927, *Procephalobus* sp. 1 WB-2008, *Propanagrolaimus* spp., and *Halicephalobus* spp.. One sequence of *Panagrolaimus paetzoldi* Goodey, 1963 isolate 452 was particularly distantly placed from the majority of *Panagrolaimus* taxa, forming a maximally supported (1.0 pp) clade with two other genera of Panagrolaimidae, *Baujardia mirabilis* Bert, De Ley, Segers, Van Driessche & De Ley, 2003 and *Panagrellus redivivus* (Linnaeus, 1767) Goodey, 1945. A different sequence identified in GenBank as *P. paetzoldi* was placed in a clade with *Procephalobus* sp. 1 WB-2008. The Panagrolaimidae family was shown to be non-monophyletic. Members of two other families, Cephalobidae (*Acrobeles complexus*) and Brevibuccidae (*Tarantobelus arachnicida* Abolafia & Peña-Santiago, 2018 and *Plectonchus wyganti* Massey, 1974) were placed in clades with members of Panagrolaimidae.

The LSU analysis produced a somewhat different topology with improved resolution (Fig. 6). *P. namibiensis* n. sp. was fully supported (1.0 pp) as sister to *P. labiatus* SN14, and that clade was strongly supported (1.0 pp) as sister to two undescribed species from Mexican soils, *Panagrolaimus* sp. HMM550 and *Panagrolaimus* sp. HMM476 (Mejía-Madrid 2018), and *Panagrolaimus* sp. S91 from an unknown location. The majority of the remaining *Panagrolaimus* species (15 out of 22) formed an additional strongly supported clade (1.0 pp) with well-resolved relationships. *P. kolymaensis* was strongly supported (1.0 pp) as sister to all but two of the other included members of *Panagrolaimus*. *Panagrolaimus* was again shown to be nonmonophyletic as *Panagrolaimus* sp. DS127 was moderately supported as part of a clade with *Turbatrix aceti*, *Halicephalobus* spp., and two members of Alloionematidae, *Rhabditophanes* sp. KR3021 and *Alloionema appendiculatum* Schneider, 1859. Members of Brevibuccidae, Alloionematidae, and one Cephalobidae formed clades with members of Panagrolaimidae, rendering the family nonmonophyletic.

### Anhydrobiosis experiment results

**Figure 7:**
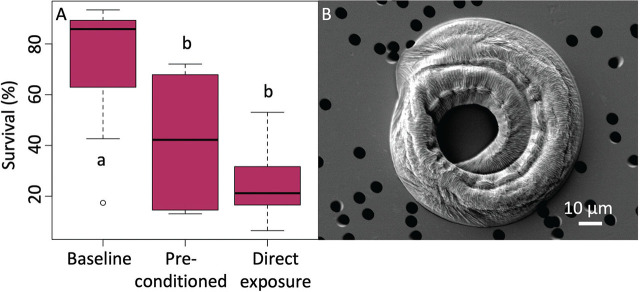
A) *Panagrolaimus namibiensis* n. sp. survival (% of population regaining motility) after exposure to three treatments. Baseline nematodes were maintained at 100% RH for 24 h. Pre-conditioned nematodes were slowly exposed to increasingly lower RH levels before 24 h at 0%. Direct exposure nematodes were directly exposed to 0% RH. Boxplots represent the interquartile range with lines representing the median value. Whiskers represent the minimum/maximum values, and circles are outliers. N = 8 replicates per treatment. Bars with different lowercase letters are statistically different from each other (Tukey’s HSD test). B) SEM of a coiled and anhydrobiotic *P. namibiensis* n. sp.

*P. namibiensis* n. sp. survived exposure to 0% RH *in vitro* (Fig. 7). Survival rates of nematodes varied among exposure treatments (ANOVA, significant treatment effect, F_2, 15_ =11.616, P = 0.0009, Fig. 7). Nematodes exposed to desiccation at 0% RH, with or without preconditioning, showed lower survival than the baseline nematodes that were not desiccated. However, there was no difference in the survival percentage between nematodes that were preconditioned and those that were not (Fig. 7).

## Discussion

Species of *Panagrolaimus* are challenging to differentiate (Abebe and Blaxter, 2003; Shatilovich et al., 2023), requiring careful integration of minute morphological features and molecular phylogenetic analyses. Here, we have described the unique morphology and phylogenetic placement for *P. namibiensis* n. sp. compared to previously described species and confirmed the novel identity of this soil nematode from the Namib Desert of Namibia, a location from which no *Panagrolaimus* spp. have previously been described. *P. namibiensis* n. sp. can be distinguished from similar species by a combination of features, including a hook-shaped stegostomal tooth and the positioning of the anterior deirids and excretory pore at mid-bulb level. Of note is the presence of a conspicuous posterior deirid that has not been reported previously in *Panagrolaimus*. This feature was observed in *P. namibiensis* n. sp. on both sides of the body on all examined nematodes where the positions were accessible via SEM. Posterior deirids have been observed in *Caenorhabditis* spp. (Slos et al. 2017; Kiontke 1997), as well as several other soil nematodes (Sturhan and Rahi, 1996). It is important to acknowledge, however, that some *Panagrolaimus* species descriptions lack morphological details (Abolafia and Peña-Santiago 2006), and that intraspecific variation in features in *Panagrolaimus* spp. has been reported (Mianowska, 1977; Boström, 1998; Yadav et al., 2023).

Phylogenetic analyses based on SSU and LSU genes each confirmed placement of *P. namibiensis* n. sp. within a clade that contained most of the species of *Panagrolaimus* included in the analyses, though each locus suggested close relationships to different taxa. Though many taxa that were shown to be closely related to *P. namibiensis* n. sp. in the SSU analysis were not able to be included in the LSU analysis (and vice versa) due to sequence availability, both analyses placed *P. namibiensis* n. sp. close to *P. labiatus* SN14. The SSU analysis placed *P. namibiensis* n. sp. as sister to *Panagrolaimus* sp. 4164 and closely related to *P. kolymaensis*. These taxa were placed in a clade similar to that recovered by the SSU analysis of Yadav et al., (2023), including *P. davidi*, *P. rigidus*, *P. labiatus*, and *P. artyukhovski*, among others. In contrast, our LSU analysis placed *P. namibiensis* n. sp. as sister to *P. labiatus* SN14 and closely related to *Panagrolaimus* sp. HMM550 and *Panagrolaimus* sp. HMM476, with the latter three taxa also having formed a clade in the LSU analysis of Yadav et al. (2023). Our LSU analysis shows improved resolution within clades of *Panagrolaimus* compared to that of Yadav et al. (2023). The topology of our LSU analysis is more similar to that resolved by the Shatilovich et al. (2023) analysis (combined SSU and LSU) than to our SSU topology, as our LSU analysis similarly placed *P. kolymaensis* outside of other members of *Panagrolaimus*. Topological comparison is difficult, however, due to little overlap in taxon inclusion between our analyses and that of Shatilovich et al. (2023). Many sequences included in the Shatilovich et al. (2023) analysis were generated by Lewis et al. (2009), and they mostly include a portion of the LSU locus that does not include the D2–D3 variable domain sequenced for our analysis and that of Yadav et al. (2023). Most LSU sequences for the taxa included in Shatilovich et al. (2023) only overlapped in the less variable 5′ half of the sequences generated by our analysis. Thus, our broader phylogenetic framework for LSU primarily came from sequences generated by Yadav et al. (2023), making comparison difficult.

There is a need for a revision of the genus *Panagrolaimus* to resolve species delineations and relationships to other genera (Andrássy 2005; Lewis et al. 2009). Our molecular phylogenetic analyses suggest the genus may be non-monophyletic, as several species (i.e., two taxa identified as *P. paetzoldi* and one taxon identified as *P. detritophagus*) fall outside the rest of *Panagrolaimus* in the SSU tree. Our LSU analysis placed just *Panagrolaimus* sp. DS127 within a clade of *Turbatrix aceti*, *Halicephalobus* spp., and members of Alloionematideae. Yadav et al. (2023) also showed *Panagrolaimus* to be non-monophyletic due solely to the distant placement of *P. paetzoldi* for both their SSU and LSU analyses. Shatilovich et al. (2023) showed *Panagrolaimus* as monophyletic, but only because they informally transferred *P. paetzoldi*, *P. detritophagus*, and several other species identified in GenBank as *Panagrolaimus* to the genus *Propanagrolaimus* because their phylogenetic analysis placed them within a clade of members of *Propanagrolaimus*. Our SSU analysis placed one of the *P. detritophagus* and one of the *P. paetzoldi* sequences in a clade with *Procephalobus* sp. 1 WB-2008.

The challenges of having different taxa sequenced for different loci or different regions of the same locus suggest that a more complete sequencing effort that includes entire LSU and SSU regions, or transcriptomes and genomes, would allow a broader understanding of the taxonomic breadth of *Panagrolaimus* and the relationships among members of the genus. SSU and LSU sequences have not been collected for all species including, for example, *P. conophthori* and *P. leperisini*. Even when available, sequences may potentially be misassigned, given the very similar morphology among *Panagrolaimus* species and other genera. A more thorough understanding of the genus would benefit from further study of topotypes of un-sequenced species, in addition to confirming the species assignments of existing sequences (*sensu* Jahanshahi Afshar, 2019).

Panagrolaimidae seems to be non-monophyletic as shown by our SSU and LSU analyses. Several members of Panagrolaimidae formed clades with members of Brevibuccidae in both our SSU and LSU analyses, and our LSU analysis showed members of the Alloionematidae nested within members of Panagrolaimidae. The analyses of Yadav et al. (2023) also show Brevibuccidae and Alloionematidae forming clades with Panagrolaimidae, rendering Panagrolaimidae non-monophyletic. Shatilovich et al. (2023) only included members of Panagrolaimidae, thus not allowing the testing of monophyly for Panagrolaimidae.

Cryptobiosis (anhydrobiosis, cryobiosis, etc.) is characterized by a suite of physiological and cellular responses that allow for survival in an ametabolic state over long periods of time when water is biologically unavailable (Adhikari et al., 2010; Tyson et al., 2012). Recent genomic analyses show that cryptobiotic nematodes possess a common genetic toolkit acquired via horizontal gene transfer that confers this survival ability (Schiffer et al., 2019; Shatilovich et al., 2023). Shatilovich et al. (2023) recently greatly expanded our understanding of the extent to which cryptobiosis can extend the nematode life span by reviving *P. kolymaensis* from 46,000-year-old mammal burrows buried in permafrost. We found that *P. namibiensis* n. sp. survived exposure to 0% RH for 24 h, with and without preconditioning at intermediate RH levels, and this nematode has the potential for future use as a model organism for the study of anhydrobiosis. In recent studies of soil nematode communities in the Namib Desert, the most common nematodes were *Panagrolaimus* spp. (Treonis et al., 2022, 2024). The dominance of this nematode under long-lived, slow-growing plants in the desert suggests that *Panagrolaimus* are uniquely suited to life in extreme environments. We observed morphological variation among *Panagrolaimus* spp. in Namib Desert soils that suggest other species may remain for future characterization.

## Conclusion

*Panagrolaimus namibiensis* n. sp. is an anhydrobiotic nematode from the Namib Desert of Namibia, one of the driest terrestrial habitats on Earth. The description, behavior in culture, and phylogenetic analysis facilitate its comparison with other *Panagrolaimus* species.

## Supplementary Table 1:

Taxon names, strain identification if available, and GenBank accession numbers of previously published SSU and LSU rDNA sequences included in the molecular phylogenetic analyses.

**Table:**

**SSU rDNA**		**LSU rDNA D2-D3**	

**Taxon Name**	**Accession Number**	**Taxon Name**	**Accession Number**
*Acrobeles complexus*	KU180671	*Acrobeles complexus*	KU180682
*Alloionema appendiculatus*	KP204844	*Alloionema appendiculatum*	KP204846
*Baldwinema ilamense*	KM454870	*Baldwinema golestanense*	KM454875
*Baujardia mirabilis*	AF547385	*Baldwinema ilamense*	KM454871
*Caenorhabditis elegans* N2	MN519140	*Caenorhabditis elegans* N2	MN519140
*Halicephalobus gingivalis*	KP875567	*Halicephalobus gingivalis*	KP875568
*Halicephalobus* sp. AA4	MF470238	*Halicephalobus* sp. AA4	MG051261
*Neoalloionema tricaudatum*	KR817916	*Panagrellus redivivus PS1163*	DQ408250
*Panagrellus redivivus* PS1163	AF083007	*Panagrobelus stammeri*	DQ145649
*Panagrobelus stammeri*	AF202153	Panagrolaimidae sp. DS129	MK087078
Panagrolaimidae sp. DS129	MK087064	*Panagrolaimus detritophagus* S136	ON927889
*Panagrolaimus* sp. ES6	FJ590961	*Panagrolaimus kolymaensis*	JAJQJQ010000137
*Panagrolaimus artyukhovskii*	MK636578	*Panagrolaimus labiatus* SN14	MZ546405
*Panagrolaimus davidi* AC_Pd2	HQ270131	*Panagrolaimus* sp. B14	OP720885
*Panagrolaimus davidi*	FJ590981	*Panagrolaimus* sp. DS127	MK087076
*Panagrolaimus detritophagus*	FJ590980	*Panagrolaimus* sp. HMM472	KY750812
*Panagrolaimus detritophagus* BS0008	EU543176	*Panagrolaimus* sp. HMM476	KY750816
*Panagrolaimus facetus*	KY119819	*Panagrolaimus* sp. HMM550	KY750827
*Panagrolaimus kolymaensis*	JAJQJQ010000793	*Panagrolaimus* sp. L73	PP786565
*Panagrolaimus labiatus* SN14	MW412913	*Panagrolaimus* sp. LA5	PP786566
*Panagrolaimus paetzoldi* 452	FJ040414	*Panagrolaimus* sp. PS1159	EU253569
*Panagrolaimus paetzoldi*	ON713642	*Panagrolaimus* sp. PS1159	DQ059061
*Panagrolaimus sp.* isolate rigidus1	MK547102	*Panagrolaimus* sp. R18	EF417146
*Panagrolaimus rigidus*	FJ590974	*Panagrolaimus* sp. RS-2007a	EF417144
*Panagrolaimus* sp. 10 ARA-2018	MH399798	*Panagrolaimus* sp. RS-2007b	EF417145
*Panagrolaimus* sp. 4164	MK301117	*Panagrolaimus* sp. S33	ON927890
*Panagrolaimus* sp. AS01	KC522708	*Panagrolaimus* sp. S71	ON927888
*Panagrolaimus* sp. DL0117	FJ590986	*Panagrolaimus* sp. S91	ON927891
*Panagrolaimus* sp. DL0128	FJ590987	*Panagrolaimus* sp. SAN-15	DQ145651
*Panagrolaimus* sp. DL0139	FJ590989	*Panagrolaimus* sp. T6_4_6	MZ425430
*Panagrolaimus* sp. ES1	FJ590960	*Plectonchus hunti*	KU180689
*Panagrolaimus* sp. ES2	FJ590962	*Rhabditophanes* sp. KR3021	AY294185
*Panagrolaimus* sp. ES3	FJ590963	*Tarantobelus arachnicida*	MZ656003
*Panagrolaimus* sp. ES5	FJ590972	*Turbatrix aceti*	KU180690
*Panagrolaimus* sp. 3 GVDU-2019	MN082330		
*Panagrolaimus* sp. JB051	FJ590971		
*Panagrolaimus* sp. JB115	FJ590969		
*Panagrolaimus* sp. JB131	FJ590968		
*Panagrolaimus* sp. JU765	FJ590956		
*Panagrolaimus* sp. PS1159	U81579		
*Panagrolaimus* sp. PS1159	FJ590977		
*Panagrolaimus* sp. PS1162	FJ590958		
*Panagrolaimus* sp. PS1579	FJ590976		
*Panagrolaimus* sp. PS1732	FJ590959		
*Panagrolaimus* sp. PS1806	FJ590957		
*Panagrolaimus* sp. PS3966	FJ590983		
*Panagrolaimus* sp. PS443	KC522712		
*Panagrolaimus* sp. PS443	FJ590978		
*Panagrolaimus* sp. SN103	FJ590970		
*Panagrolaimus subelongatus*	AY284681		
*Panagrolaimus superbus*	KC522707		
*Panagrolaimus superbus*	FJ590973		
*Panagrolaimus trilabiatus*	KF011487		
*Plectonchus wyganti*	KJ636307		
*Procephalobus* sp. 1 WB-2008	EU543179		
*Propanagrolaimus* sp. LC91	KJ434175		
*Propanagrolaimus* sp. WTM1	KJ434176		
*Rhabditophanes* sp. KR3021	AF202151		
*Strongyloides procyonis*	AB205054		
*Tarantobelus arachnicida*	MZ655999		
*Turbatrix aceti*	KU180673		
